# Antibiotics Enhance Prevention and Eradication Efficacy of Cathodic-Voltage-Controlled Electrical Stimulation against Titanium-Associated Methicillin-Resistant Staphylococcus aureus and Pseudomonas aeruginosa Biofilms

**DOI:** 10.1128/mSphere.00178-19

**Published:** 2019-05-01

**Authors:** Mary K. Canty, Lisa A. Hansen, Menachem Tobias, Sandy Spencer, Terry Henry, Nicole R. Luke-Marshall, Anthony A. Campagnari, Mark T. Ehrensberger

**Affiliations:** a Department of Biomedical Engineering, University at Buffalo, Buffalo, New York, USA; b Department of Microbiology and Immunology, University at Buffalo, Buffalo, New York, USA; c Department of Orthopaedics, University at Buffalo, Buffalo, New York, USA; Centers for Disease Control and Prevention

**Keywords:** orthopedic infection, treatment, prevention, bacteria, biofilms

## Abstract

Periprosthetic joint infection (PJI) develops clinically, even with antibiotic treatment, and methicillin-resistant Staphylococcus aureus (MRSA) and Pseudomonas aeruginosa are predominant causes of these infections. Due to biofilm formation, antibiotic treatment for patients with PJI can perpetuate resistance, further complicating the use of noninvasive treatments. This study evaluated cathodic-voltage-controlled electrical stimulation (CVCES) of titanium, in combination with a clinically relevant antibiotic, to synergistically prevent MRSA and P. aeruginosa PJIs by inhibiting bacterial adherence or as a treatment for eradicating established biofilms. CVCES of −1.0 V, −1.5 V, or −1.8 V (versus Ag/AgCl), with or without vancomycin for MRSA or gentamicin for P. aeruginosa, was applied to sterile titanium incubated with cultures to evaluate prevention of attachment or eradication of preestablished biofilms. Treatments were 24 h long and included open-circuit potential controls, antibiotic alone, CVCES, and CVCES plus antibiotic. Biofilm-associated and planktonic CFU were enumerated. In general, CVCES at −1.8 V alone or with antibiotic completely eradicated biofilm-associated CFU for both strains, and these parameters were also highly effective against planktonic bacteria, resulting in a >6-log reduction in MRSA and no detectable planktonic P. aeruginosa. All CFU were reduced ∼3 to 5 logs from controls for prevention CVCES plus antibiotics at −1.0 V and −1.5 V against MRSA. Remarkably, there were no detectable P. aeruginosa CFU following prevention CVCES at −1.0 V or −1.5 V with gentamicin. Our results suggest that CVCES in combination with antibiotics may be an effective approach for prevention and treatment of PJI.

**IMPORTANCE** Periprosthetic joint infections (PJIs) develop clinically in the presence of antibiotic therapies and are responsible for increased patient morbidity and rising health care costs. Many of these infections involve bacterial biofilm formation on orthopedic hardware, and it has been well established that these biofilms are refractory to most antibiotic treatments. Recent studies have focused on novel methods to prevent and eradicate infection. Cathodic-voltage-controlled electrical stimulation (CVCES) has previously been shown to be effective as a method for prevention and eradication of Gram-positive and Gram-negative infections. The present study revealed that the utility of CVCES for prevention and eradication of methicillin-resistant Staphylococcus aureus and Pseudomonas aeruginosa is enhanced in the presence of clinically relevant antibiotics. The synergistic effects of CVCES and antibiotics are effective in a magnitude-dependent manner. The results of this study indicate a promising alternative method to current PJI mitigation techniques.

## Abstract (precis)

Periprosthetic joint infections (PJIs) develop clinically in the presence of antibiotic therapies and are responsible for increased patient morbidity and rising health care costs. Many of these infections involve bacterial biofilm formation on orthopedic hardware, and it has been well established that these biofilms are refractory to most antibiotic treatments. Recent studies have focused on novel methods to prevent and eradicate infection. Cathodic-voltage-controlled electrical stimulation (CVCES) has previously been shown to be effective as a method for prevention and eradication of Gram-positive and Gram-negative infections. The present study revealed that the utility of CVCES for prevention and eradication of methicillin-resistant Staphylococcus aureus and Pseudomonas aeruginosa is enhanced in the presence of clinically relevant antibiotics. The synergistic effects of CVCES and antibiotics are effective in a magnitude-dependent manner. The results of this study indicate a promising alternative method to current PJI mitigation techniques.

## INTRODUCTION

It is projected that by 2030, the number of primary total hip arthroplasty (THA) and total knee arthroplasty (TKA) procedures performed annually in the United States will increase by 174% and 673%, respectively (1). Over the same time period, the incidence of periprosthetic joint infections (PJIs) following TKA and THA is projected to increase to 6.8% (2), necessitating the treatment of approximately 272,000 patients for PJI annually. The economic burden is expected to exceed $55 billion (2).

One of the primary mechanisms by which bacteria are thought to resist decontamination and persist as chronic infections on orthopedic devices is through the formation of biofilms. Bacteria in biofilms can be 500 to 5,000 times more resistant to antibiotic therapy than their planktonic counterparts (3). As a result, antibiotic treatments have little to no effect on the bacteria in a biofilm, making these infections extremely difficult to eradicate. Gram-positive methicillin-resistant Staphylococcus aureus (MRSA) and Gram-negative Pseudomonas aeruginosa are two problematic pathogens of increasing concern due to their multidrug resistance (4, 5). These species are commonly found in life-threatening nosocomial infections, including PJI (6), creating serious and potentially untreatable clinical complications. Unfortunately, as the incidence of multidrug-resistant pathogens rises, the number of successful antibiotics on the market is diminishing. Recent reports indicate that the development of successful antibacterial drugs on the market in the United States will continue to stagnate over the coming years (4), increasing the importance of developing alternative methods of prevention and treatment of biofilm-associated infections.

Traditionally, β-lactam antibiotics are used in orthopedics for surgical prophylaxis (7), and while prophylaxis with cefazolin is most common, combined treatment with gentamicin and cefazolin or treatment with gentamicin alone has also been documented (8, 9). Additionally, in cases of β-lactam allergy or when a patient is colonized with MRSA, vancomycin prophylaxis is used (10). However, the growing concern over antimicrobial resistance has heightened interest in discovering alternative methods to prevent and eradicate PJI.

While the goal of surgical antimicrobial prophylaxis is to prevent PJI, infection can still develop and often requires an additional course of long-term antibiotics (11). Persistent PJIs necessitate recurrent surgical debridement and often removal of the orthopedic hardware in an attempt to aggressively eradicate the infection (12). The current gold-standard treatment for PJI is a two-stage revision. The first stage involves thorough irrigation and debridement of the surrounding tissues, exchange of the infected implant for an antibiotic-laden bone cement spacer, and a prolonged course of systemic antibiotics. This process leaves the patient biomechanically deficient until the infection is cleared and a new prosthesis can be implanted. Unfortunately, in roughly 25% of PJI patients, this treatment is ineffective (10). These persistent and refractory infections often require more drastic measures, such as joint fusion or limb amputation, for definitive treatment. Disturbingly, PJI is also associated with increased mortality, with only an 87% 5-year survival rate (13).

Titanium is commonly utilized in orthopedic and dental applications due to its high mechanical strength and excellent biocompatibility. A spontaneously forming surface oxide layer passivates the surface and provides titanium with excellent corrosion resistance. Previously, it has been shown that the faradaic and nonfaradaic electrochemical properties of commercially pure titanium (cpTi) are voltage dependent (14–19) and contribute to the electrochemical prevention and removal of biofilms.

Previous studies have shown that cathodic-voltage-controlled electrical stimulation (CVCES) of cpTi is a promising antimicrobial strategy to prevent and eradicate implant-associated MRSA infections (15, 18, 20, 21). To further investigate the potential of CVCES as a novel therapeutic for both the prevention of PJI development and the eradication of established PJIs due to multidrug-resistant pathogens, the present *in vitro* study evaluated the antimicrobial efficacy of CVCES at various magnitudes in combination with antibiotic therapy against MRSA and P. aeruginosa.

## RESULTS

### Eradication of bacterial biofilms using CVCES in combination with antibiotics.

**FIG 1:**
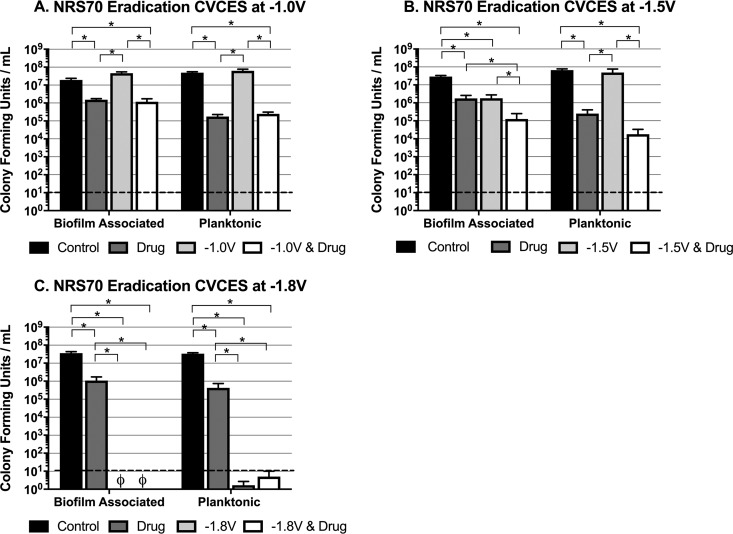
Eradication of established MRSA strain NRS70 biofilms on cpTi after 24 h of CVCES in the presence or absence of vancomycin. Experiments were conducted at −1.0 V (A), −1.5 V (B), and −1.8 V (C). The conditions reported are for incubations with no stimulation (control), vancomycin alone (drug), CVCES alone (−1.0 V, −1.5 V, or −1.8 V), or CVCES with vancomycin (−1.0 V, −1.5 V, or −1.8 V plus drug). ø indicates that no detectable CFU were enumerated. Bar plots show mean values ± 1 standard deviation calculated from the four independent samples under each condition. * indicates statistically significant differences between the groups as specified. The limit of detection is denoted with a dashed line.

Established biofilms of MRSA strain NRS70 and P. aeruginosa strain PA27853 preformed on cpTi coupons were subjected to CVCES at −1.0 V, −1.5 V, and −1.8 V for a duration of 24 h, in the presence and absence of antibiotics, followed by assessment of bacterial viability posttreatment. All voltages are reported with respect to a chlorided silver wire (Ag/AgCl) reference electrode. The results of the eradication experiments evaluating the effects of single and combination treatments on NRS70 are shown in Fig. 1. For the −1.0-V CVCES data set, only experimental treatments containing vancomycin significantly reduced biofilm-associated and planktonic NRS70, indicating that the observed reductions are solely antibiotic mediated (Fig. 1A). CVCES alone at −1.5 V significantly reduced biofilm-associated NRS70 but had no effect on planktonic bacteria (Fig. 1B). However, CVCES at −1.5 V in combination with vancomycin produced significant and synergistic reductions in biofilm-associated NRS70 compared to all other experimental conditions. This combined treatment also showed significant reductions in planktonic NRS70 compared to controls and CVCES alone. Figure 1C shows that there were no viable biofilm-associated or planktonic NRS70 detected after CVCES at −1.8 V in both the presence and absence of vancomycin.

**FIG 2:**
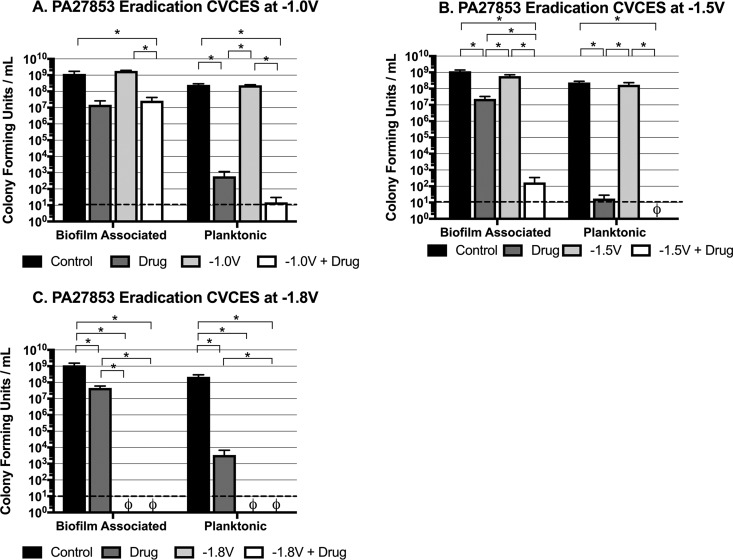
Eradication of established P. aeruginosa strain PA27853 biofilms on cpTi after 24 h of CVCES in the presence or absence of gentamicin. Experiments were conducted with CVCES at −1.0 V (A), −1.5 V (B), and −1.8 V (C). The conditions reported are for incubations with no stimulation (control), gentamicin alone (drug), CVCES alone (−1.0 V, −1.5 V, or −1.8V), or CVCES with gentamicin (−1.0 V, −1.5 V, or −1.8 V plus drug). ø indicates that no detectable CFU were enumerated under the test condition. Bar plots show mean values ± 1 standard deviation calculated from the four independent samples under each condition. * indicates a statistically significant differences between the groups as specified. The limit of detection is denoted with a dashed line.

Given the many differences in membrane structure between Gram-positive and Gram-negative bacteria, these CVCES parameters were further evaluated for efficacy against established biofilms of the P. aeruginosa clinical isolate PA27853 in the presence and absence of gentamicin (Fig. 2). Under the −1.0-V condition (Fig. 2A), only treatments in the presence of gentamicin reduced biofilm-associated and planktonic CFU at least 2 logs from controls. While −1.5-V CVCES alone had no significant effect on biofilm-associated or planktonic PA27853, combination treatment with gentamicin showed biologically significant reductions in biofilm-associated and planktonic PA27853 of 7 logs and 8 logs, respectively (Fig. 2B). It is further noteworthy to emphasize that combining this CVCES condition with antibiotic treatment produced synergistic reductions in biofilm-associated CFU that were significantly more robust than either of the treatments applied individually. After 24 h of CVCES at −1.8 V, there were no detectable PA27853 bacteria in both the presence and absence of gentamicin (Fig. 2C), which was consistent with the results seen for NRS70.

### Eradication CVCES affects medium alkalinity.

Previous reports have illustrated the relationship between microenvironment pH by electrical stimulation and its effects on bacterial viability (22–25). Most bacterial species have specific pH ranges (range of 2 to 3 pH units) in which growth is possible (26), and forcing the pH of the surrounding environment outside acceptable levels can induce bactericidal effects. Therefore, for all of the eradication experiments described above, pH measurements for the medium after 24 h of treatment were recorded (Table 1). For each set of NRS70 and PA27853 parameters, the pH of the medium exposed to CVCES, either alone or in combination with antibiotics, increased as the applied cathodic potential increased, reaching a maximum pH of ∼12.5 with CVCES at −1.8 V. In contrast, the pH of the medium exposed to relevant controls remained fairly constant.

**TABLE 1:** pH measured following eradication CVCES for NRS70 and PA27853

Parameter^*a*^	Value for strain					
	NRS70			PA27853		
	−1.0 V	−1.5 V	−1.8 V	−1.0 V	−1.5 V	−1.8 V
Mean pH ± SD under condition:						
A	5.68 ± 0.09	5.35 ± 0.32	5.42 ± 0.24	7.43 ± 0.07	7.41 ± 0.06	7.49 ± 0.24
B	7.17 ± 0.22	6.90 ± 0.22	6.73 ± 0.14	6.73 ± 0.07	6.66 ± 0.03	6.80 ± 0.07
C	5.97 ± 0.24	9.13 ± 1.1	12.4 ± 0.24	7.51 ± 0.14	9.17 ± 0.13	12.6 ± 0.11
D	7.84 ± 0.07	10.3 ± 0.43	12.5 ± 0.26	7.18 ± 0.19	9.70 ± 0.76	12.5 ± 0.28

*P* value						
A vs B	<0.001	<0.001	<0.001	<0.001	<0.001	0.002
A vs C	0.111	0.001	<0.001	0.860	<0.001	<0.001
A vs D	<0.001	<0.001	<0.001	0.061	0.027	<0.001
B vs C	<0.001	0.013	<0.001	<0.001	<0.001	<0.001
B vs D	0.001	<0.001	<0.001	0.001	0.012	<0.001
C vs D	<0.001	0.208	0.704	0.015	0.586	0.823

a A, control; B, drug; C, CVCES; D, CVCES plus drug.

### Cathodic current density and charge transfer calculated from eradication CVCES.

The current density is a function of the applied potential and gives information about the rate of the reactions at the electrode-electrolyte interface. At the potentials studied here, the current density indicates the rates of the oxygen and water reduction reactions. In general, the cathodic current density and cumulative charge transfers measured during the eradication experiments were shown to increase at higher cathodic potentials under all test conditions with exposure to CVCES treatment (Table 2), and these increases were all significant.

**TABLE 2:** Cathodic current density and cumulative charge transfer calculated from eradication CVCES

Parameter^*a*^	Cathodic current density (μA/cm^2^)		Cumulative charge transfer (coulomb)	
	CVCES	CVCES + drug	CVCES	CVCES + drug
NRS70				
Mean value ± SD under condition:				
A	−3.26 ± 0.97	−5.67 ± 0.72	1.23 ± 0.36	2.06 ± 0.27
B	−34.1 ± 4.31	−42.4 ± 8.50	12.8 ± 1.62	16.0 ± 3.20
C	−749 ± 281	−1248 ± 351	282 ± 106	470 ± 132
*P* value				
A vs B	0.004	0.003	<0.001	<0.001
B vs C	<0.001	<0.001	<0.001	<0.001
A vs C	<0.003	0.003	<0.001	<0.001

PA27853				
Mean value ± SD under condition:				
D	−2.11 ± 1.52	−3.85 ± 1.03	0.79 ± 0.57	1.45 ± 0.39
E	−26.2 ± 5.35	−52.0 ± 31.0	8.28 ± 4.59	19.6 ± 11.7
F	−1,156 ± 76.8	−1,773 ± 994	668 ± 374	436 ± 28.9
*P* value				
D vs E	0.011	<0.001	0.004	<0.001
E vs F	<0.001	<0.001	<0.001	<0.001
D vs F	0.001	<0.001	<0.001	<0.001

a A, −1.0 V; B, −1.5 V; C, −1.8 V; D, −1.0 V; E, −1.5 V; F, −1.8 V.

### CVCES with antibiotics prevents bacterial growth and biofilm formation.

**FIG 3:**
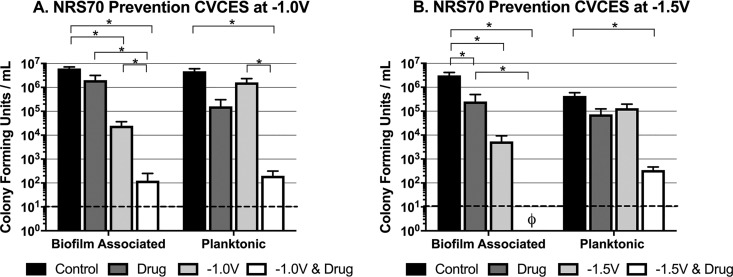
Prevention of MRSA strain NRS70 growth and biofilm formation on cpTi after 24 h of CVCES in the presence or absence of vancomycin. Experiments were conducted with CVCES at −1.0 V (A) or −1.5 V (B). The conditions reported are for incubations with no stimulation (control), vancomycin alone (drug), CVCES alone (−1.0 V or −1.5 V), or CVCES with vancomycin (−1.0 V or −1.5 V plus drug). ø indicates that no detectable CFU were enumerated under the test condition. Bar plots show mean values ±1 standard deviation calculated from the four independent samples under each condition. * indicates statistically significant differences between the groups as specified. The limit of detection is denoted with a dashed line.

In this series of studies, CVCES was further evaluated as a single treatment and in combination with relevant antibiotics for the ability to prevent bacterial growth, attachment, and biofilm formation on cpTi. To evaluate prevention efficacy, freshly inoculated wells containing ∼10^3^ CFU/ml of logarithmically growing bacteria and a sterile cpTi coupon were exposed for 24 h to CVCES at −1.0 V or −1.5 V in the presence or absence of antibiotics. Subsequently, CFU were enumerated for planktonic bacteria in the medium and biofilm-associated bacteria adherent to the submerged cpTi coupon. An evaluation of CVCES at −1.8 V for 24 h was not performed due to previous prevention studies showing that the application of CVCES alone at −1.8 V for 8 h had a sterilizing antimicrobial effect (15). Figure 3 shows the set of prevention experiments conducted with NRS70 in the presence or absence of vancomycin. Compared to controls, CVCES alone at −1.0 V (Fig. 3A) and −1.5 V (Fig. 3B) significantly reduced the biofilm-associated CFU but had little effect on the planktonic bacteria. In contrast, the combination of −1.5-V CVCES with vancomycin completely prevented bacterial colonization and subsequent biofilm formation on the cpTi coupon and produced an additional 2-log reduction in planktonic NRS70 compared to the single-treatment controls alone.

**FIG 4:**
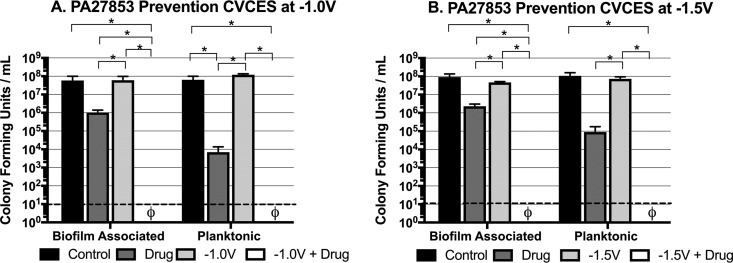
Prevention of P. aeruginosa strain PA27853 growth and biofilm formation on cpTi after 24 h of CVCES in the presence or absence of gentamicin. Experiments were conducted with CVCES at −1.0 V (A) or −1.5 V (B). The conditions reported are for incubations with no stimulation (control), gentamicin alone (drug), CVCES alone (−1.0 V or −1.5 V), or CVCES with gentamicin (−1.0 V or −1.5 V plus drug). ø indicates that no detectable CFU were enumerated under the test condition. Bar plots show mean values ± 1 standard deviation calculated from the four independent samples under each condition. * indicates statistically significant differences between the groups as specified. The limit of detection is denoted with a dashed line.

These prevention studies were repeated against PA27853. Figure 4 depicts biofilm-associated and planktonic PA27853 CFU enumerated for the tests conducted at −1.0 V (Fig. 4A) and −1.5 V (Fig. 4B). Combination treatments evaluating gentamicin with CVCES at −1.0 V (Fig. 4A) and −1.5 V (Fig. 4B) resulted in complete inhibition of any detectable biofilm-associated bacteria adherent to the cpTi coupon and complete killing of planktonic PA27853. The reductions in biofilm-associated CFU for the combined treatments were again shown to be synergistic, as they had significantly greater antimicrobial effects than either of the individual treatments.

### Prevention CVCES affects medium alkalinity.

Table 3 shows pH measurements taken after 24 h of incubation in the presence or absence of vancomycin (NRS70) and gentamicin (PA27853) for the set of prevention experiments conducted with CVCES at −1.0 V and −1.5 V. The data clearly show that exposure to CVCES produced an alkaline shift in pH that increased with the magnitude of the CVCES applied.

**TABLE 3:** pH measured following prevention CVCES for NRS70 and PA27853

Parameter^*a*^	Value for strain			
	NRS70		PA27853	
	−1.0 V	−1.5 V	−1.0 V	−1.5 V
Mean pH ± SD under condition:				
A	6.83 ± 0.13	6.99 ± 0.07	7.14 ± 0.14	7.08 ± 0.07
B	7.07 ± 0.05	7.03 ± 0.09	7.02 ± 0.04	7.06 ± 0.045
C	7.74 ± 0.25	9.96 ± 0.54	7.35 ± 0.29	9.17 ± 0.087
D	8.00 ± 0.23	10.0 ± 0.38	8.07 ± 0.13	9.51 ± 0.11

*P* value				
A vs B	0.321	0.808	0.475	0.994
A vs C	<0.001	0.000	0.587	<0.001
A vs D	<0.001	<0.001	<0.001	<0.001
B vs C	0.001	0.001	0.275	<0.001
B vs D	<0.001	<0.001	<0.001	<0.001
C vs D	0.238	0.956	0.032	<0.001

a A, control; B, drug; C, CVCES; D, CVCES plus drug.

### Cathodic current density and charge transfer calculated from prevention CVCES.

Cathodic current densities and cumulative charge transfer values determined from prevention experiments at −1.0 V and −1.5 V are presented in Table 4. Significant differences were reported across stimulation magnitudes for both the current density and cumulative charge transfer.

**TABLE 4:** Cathodic current density and cumulative charge transfer calculated from prevention CVCES

Parameter^*a*^	Cathodic current density (μA/cm^2^)		Cumulative charge transfer (coulomb)	
	CVCES	CVCES + drug	CVCES	CVCES + drug
NRS70				
Mean value ± SD under condition:				
A	−5.51 ± 0.94	−5.64 ± 1.01	2.07 ± 0.35	2.13 ± 0.38
B	−51.3 ± 34.4	−36.5 ± 8.5	19.3 ± 12.9	13.7 ± 3.20
*P* value for A vs B	<0.001	<0.001	<0.001	<0.001

PA27853				
Mean value ± SD under condition:				
C	−3.7 ± 0.3	−5.5 ± 0.70	1.37 ± 0.12	2.09 ± 0.25
D	−25.8 ± 1.27	−27.9 ± 3.51	9.73 ± 0.47	10.5 ± 1.32
*P* value for C vs D	<0.001	<0.001	<0.001	<0.001

a A, −1.0 V; B, −1.5 V; C, −1.0 V; D, −1.5 V.

### Effect of pH on planktonic and biofilm-associated NRS70 and PA27853.

**FIG 5:**
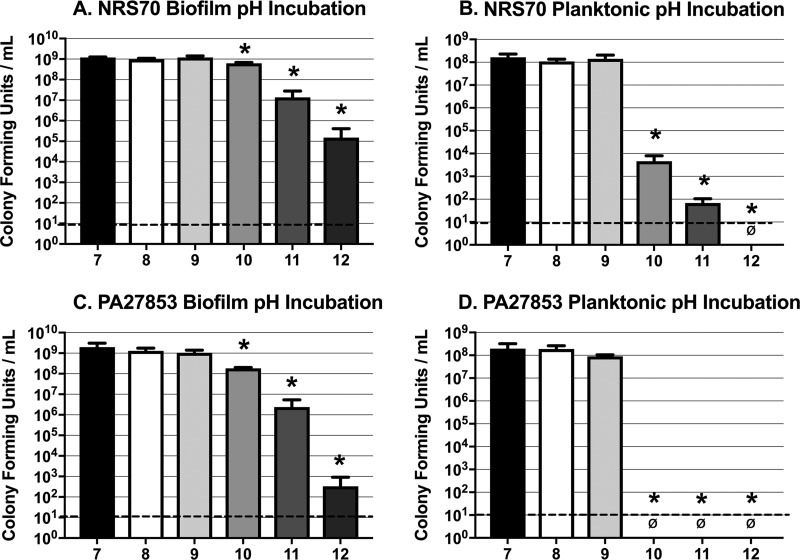
Survival of MRSA strain NRS70 and P. aeruginosa strain PA27853 at alkaline pH after 24 h of exposure. Shown are CFU for NRS70 biofilm (A) and planktonic cultures (B) and PA27853 biofilm (C) and planktonic cultures (D) following exposure to medium adjusted to a pH ranging from 7 to 12 for 24 h. ø indicates that no detectable CFU were enumerated under the test condition. A minimum of 3 independent replicates were performed under each condition. The limit of detection is denoted with a dashed line. * indicates statistically significant differences between the groups specified and the pH 7 condition.

Due to the suspected correlation between increased pH and decreased bacterial viability associated with CVCES, these studies were designed to determine the relationship between alkaline microenvironment pH and bactericidal effects. Here, planktonic cultures and established biofilms of NRS70 and PA27853 were exposed to medium that was chemically titrated to a range of alkaline pH values. Figure 5 shows the results of incubating planktonic cultures and biofilms for 24 h in medium chemically titrated to pH 7, 8, 9, 10, 11, or 12. These data demonstrate that the NRS70 biofilms (Fig. 5A) do not show any decline in viability until pH 10 and reach an ∼4-log reduction in CFU at pH 12. In contrast, planktonic NRS70 bacteria (Fig. 5B) are more sensitive to pH, as there is an ∼4-log reduction in CFU once the pH increases to 10. There was a modest sensitivity of PA27853 biofilms (Fig. 5C) to pH 10 and a more pronounced ∼3-log decline in PA27853 biofilm viability at pH 11, which increased to an ∼7-log decline at pH 12. The PA27853 planktonic cultures (Fig. 5D) were clearly more sensitive than the biofilms, as there were no planktonic CFU detected when the medium was titrated at or above pH 10.

## DISCUSSION

The emergence of multidrug-resistant pathogens necessitates alternative prevention and eradication methods for PJI (10, 27, 28). MRSA and P. aeruginosa are problematic pathogens in orthopedics that have developed clinically in the presence of prophylactic surgical antibiotics and can be difficult to treat with traditional irrigation, debridement, and antimicrobial therapy. Our results align with the clinical observation that antibiotic treatment alone can be ineffective at infection eradication and prevention (29, 30). Notably, the outcomes of this study reveal that applying CVCES directly to cpTi for 24 h can be effective for eradicating or preventing an associated MRSA or P. aeruginosa bacterial burden and, significantly, that the bactericidal effects of CVCES can be synergistically enhanced by concurrent antibiotic treatment.

The eradication studies reported here showed that extended-duration (24-h) CVCES had more robust antimicrobial effects than in previously reported eradication studies that utilized shorter-duration (1-h) CVCES (18). Importantly, it was shown that CVCES at −1.8 V for 24 h effectively eradicated biofilm-associated and planktonic NRS70 bacterial burdens. In addition, for the first time, the present study also shows the efficacy of CVCES for eradicating Gram-negative biofilms on cpTi using P. aeruginosa as the model organism. CVCES at −1.8 V completely eradicated planktonic and biofilm-associated PA27853 after 24 h. For both organisms, the effects of CVCES alone were magnitude dependent, with higher cathodic potentials exhibiting greater reductions in viability.

Additionally, this work also expanded upon previous, shorter-duration, CVCES prevention studies (15) by extending the stimulation duration to 24 h. This extended exposure to CVCES showed 2- to 3-log reductions in biofilm-associated NRS70 after CVCES treatment at −1.0 V and −1.5 V. Interestingly, these effects were not seen for PA27853, where CVCES alone at −1.0 V and −1.5 V had no effects on biofilm or planktonic bacteria after 24 h. It is possible that the variations in the cell wall compositions of Gram-positive MRSA and Gram-negative P. aeruginosa are important factors that influence the effectiveness of CVCES alone to prevent bacterial colonization of the cpTi surfaces.

Our eradication and prevention studies utilized cpTi because titanium and its alloys are frequently used in orthopedic and dental applications due to their excellent biocompatibility, high strength, and osseointegration qualities. When CVCES is applied to cpTi in the presence or absence of antibiotics, significant increases in the pH were observed compared to the no-treatment controls (Tables 1 and 3). This increase is likely due to the production of hydroxide ions as a result of the water and oxygen reduction reactions occurring on the cpTi surface. The results of our study also indicate that higher cathodic current density, and, consequentially, greater cumulative charge transfer, occurs when higher magnitudes of CVCES were applied to cpTi (Tables 2 and 4). These larger faradaic processes were also associated with greater alkaline pH shifts of the microenvironment and more effective prevention and eradication of NRS70 or PA27853 from the surface of cpTi.

The observations from the present study, combined with previously reported CVCES outcomes (15), suggested that pH was related to the antimicrobial effects of CVCES in an exposure-time- and pH-dependent manner. The results implied that modest alkaline pH values (∼8 to 9) need extended durations (24 h) to be antimicrobial, whereas higher alkaline pH values (∼12) can be antimicrobial at shorter durations (4 to 8 h) (15). However, the effect of alkaline medium pH was further evaluated in the absence of CVCES and revealed some discrepancies between the antimicrobial effects of electrochemically generated alkaline medium pH and chemically titrated alkaline medium pH. For example, reductions in the viability of established NRS70 and PA27853 biofilms or planktonic cultures were noted only when the chemically titrated medium pH was at or above pH 10, whereas antimicrobial effects were shown at more modest electrochemically generated alkaline pH values. Furthermore, exposure to chemically titrated alkaline medium at pH 12 was unable to completely eradicate the biofilms, but complete biofilm eradication was noted when CVCES at −1.8 V electrochemically generated an alkaline medium pH of 12. These discrepancies highlight that alkaline pH alone is not solely responsible for the antimicrobial effects and that other mechanisms associated with the CVCES electrochemical processes are also likely contributing to the antimicrobial outcomes. For example, others have shown that H_2_O_2_ can be electrochemically generated at cathode surfaces and can contribute to bactericidal activity (31–35). Additionally, others have suggested H_2_ gas evolution as the primary mechanism responsible for the removal of a 10-day-old P. aeruginosa biofilm preformed on 316L stainless steel substrates polarized to magnitudes cathodic to −1.5 V versus Ag/AgCl (36). It is possible that H_2_ gas is produced in the −1.8-V CVCES and acts concurrently with the basic pH to mechanically disrupt the biofilm to create these differences. Another aspect to consider is that pH measurements reported in the present CVCES study were bulk pH measurements made only at the end of the 24-h tests. Therefore, the temporal and spatial profile of how the medium pH changes during the tests or how long the bacteria are exposed to these electrochemically generated alkaline pH levels is unknown. Future work will address this knowledge gap and measure the change in pH as a function of CVCES over time.

The prevention studies showed that combining CVCES with concurrent antibiotic therapy provides synergistic reductions in the number of bacteria that attach to the cpTi for both NRS70 and PA27853. Furthermore, synergistic reductions in the preformed biofilms of NRS70 and PA27853 were also evident in the eradication experiments that combined CVCES at −1.5 V with antibiotics. The synergism between electrical stimulation and antimicrobial agents has been previously documented (20, 21, 33, 37–44). Of particular relevance, antimicrobial synergy has been shown for electrical stimulation combined with vancomycin against S. aureus (39) and with tobramycin against P. aeruginosa (33, 40–42, 44). However, there are important differences to highlight in the experimental protocols between the present study and those previous studies. Many of those previous studies assessed the effects of electrical stimulation by bringing the stimulating electrodes in proximity to biofilms formed on the surface of other materials (33, 39, 40, 44). In the present study, the cpTi coupons on which bacterial interactions were assessed were also utilized as the stimulating electrode. This emphasizes the role of interfacial electrochemistry in our study and importantly mimics what would be done clinically if electrical stimulation was applied directly to metallic implants for infection control. Furthermore, many previous studies have utilized current-controlled electrical stimulation delivered via a two-electrode configuration that exposed bacteria to both oxidation and reduction processes (39–42, 44), whereas the present study utilized three-electrode CVCES to assess antimicrobial effects of isolated reduction processes. Furthermore, in those previous reports, it was often necessary to use antibiotic concentrations that were well above the MIC (5-fold to 20-fold higher) to show synergy between electrical stimulation and antibiotics (33, 39, 40, 44). It is important to note that our studies used the MIC of each antibiotic, which resulted in a remarkable synergy in combination with CVCES. This is a significant observation given that the overuse of antibiotics is a major cause of the development of resistance. Although the MIC does not always correlate with the concentrations used in patients, it represents a conservative and translatable value to study the synergistic effects of CVCES with antibiotics. These encouraging results indicate that future studies are warranted to determine if our CVCES parameters in combination with other relevant antibiotics are as effective against a much broader range of organisms that are also associated with PJI.

Another interesting aspect of both the prevention and eradication studies was that a similar alkaline pH (∼9 to 10) was achieved when CVCES of −1.5 V was applied in both the absence and presence of the antibiotic; however, synergistic reductions in biofilm-associated CFU were reported for the combined treatment. In addition, in the prevention study, CVCES at −1.0 V, in both the presence and absence of the antibiotic, resulted in pH ∼8, but again, synergistic reductions in both biofilm-associated and planktonic CFU occurred with the combined treatment. This may indicate that the alkaline pH, either alone or in combination with other CVCES-associated electrochemical processes, modifies the antibiotics to enhance their effectiveness and/or alters the physiology of the bacteria or the structure of the biofilm, rendering it more susceptible to the antibiotic.

It has previously been shown that pH can alter biofilm structure and that biofilms can expand in thickness upon cathodic polarization of platinum substrates (43). Furthermore, polarized electrodes may disrupt the charge distribution within a biofilm and enhance the transport mechanism of charged molecules, such as antimicrobial agents, through the diffusion barrier of the biofilm matrix (37–39). Others have shown that significant alterations in membrane permeability, membrane potential, and intracellular reactive oxygen species occur in bacteria upon exposure to electrical stimulation (33, 42) and altered environmental pH (45). Furthermore, it has been documented that aminoglycoside uptake, and specifically gentamicin uptake, is heavily dependent on the transmembrane potential (46–49). Therefore, in the present study, it is hypothesized that the application of cathodic polarization to the cpTi may have altered the transmembrane potential of PA27853 to enhance the uptake of gentamicin compared to treatment in the absence of electrical stimulation. This also supports our finding that there was an 8-log difference in recoverable biofilm-associated and planktonic CFU with CVCES combined with gentamicin compared to CVCES alone.

In summary, we evaluated the bactericidal efficacy of CVCES at multiple levels in the presence and absence of antibiotics. CVCES at −1.8 V for 24 h showed eradication of biofilm-associated and planktonic bacteria below detectable levels for both MRSA and P. aeruginosa. While encouraging, these parameters may be restricted depending on the overall size and material of the implant. In addition, the use of CVCES in a clinical setting would most likely require the use of antibiotics to guard against possible systemic spread of bacteria that are released from biofilms. Thus, we further evaluated CVCES at lower magnitudes for longer durations in combination with relevant antibiotics to determine if this technology could be more broadly applicable. These studies confirmed that CVCES at −1.5 V for 24 h in combination with antibiotic therapy results in synergistic and significant bactericidal activity in both the eradication and prevention studies. Remarkably, CVCES at −1.5 V with antibiotic prophylaxis was able to prevent MRSA and P. aeruginosa attachment on the cpTi coupon from reaching detectable levels. Furthermore, prevention CVCES at −1.0 V in combination with vancomycin prophylaxis was able to synergistically reduce both the biofilm-associated and planktonic MRSA bacterial burdens to approximately 10^2^ CFU/ml, and the same treatment with P. aeruginosa reduced viable CFU to levels below detection.

This work shows promising future applications of CVCES for the prevention and eradication of PJI. Clinically, this CVCES technology could potentially be used for infection prevention immediately following implantation while the patient is in recovery or, in the event of PJI, could be used in lieu of a 2-stage exchange joint arthroplasty. More research is required to determine the histological effects of the extended duration of an applied voltage *in vivo*; however, previous *in vivo* studies have revealed that exposure to −1.8-V CVCES for 1 h in an *in vivo* rodent shoulder model had no deleterious histological effects on the bone or surrounding tissue following stimulation (18, 20, 21). Future considerations for human use include, but are not limited to, the size of the implant, the total current, as well as the implant material. The exact mechanisms governing the antimicrobial effects of this technology are unknown but are postulated to include a CVCES-induced faradaic modification of the surrounding microenvironment. Continued optimization of the CVCES parameters in combination with clinically relevant antibiotic therapies may provide novel and effective strategies to prevent and or eradicate PJI postimplantation.

## MATERIALS AND METHODS

### Bacterial strains.

Experiments were conducted using the Gram-positive bacterium MRSA, strain NRS70, a clinical respiratory isolate (50), or the Gram-negative bacterium P. aeruginosa, ATCC type strain PA27853, a clinical blood isolate. Bacteria were routinely cultured in tryptic soy broth supplemented with 0.25% glucose (TSBG) for NRS70 or Mueller-Hinton (MH) broth for PA27853 and grown at 37°C with aeration or plated on MH agar plates and incubated overnight at 37°C in 5% CO_2_ (both NRS70 and PA27853).

### Coupon preparation.

Commercially pure titanium (cpTi) (grade 2; Titanium Industries, Inc.) metal coupons (0.68 cm long by 0.68 cm wide by 1.25 cm high) were wet sanded to a 600-grit finish, sonicated in deionized water for 10 min, submerged in 70% ethanol for 20 min, and sterilized under UV light for 30 min.

### Chamber preparation.

**FIG 6:**
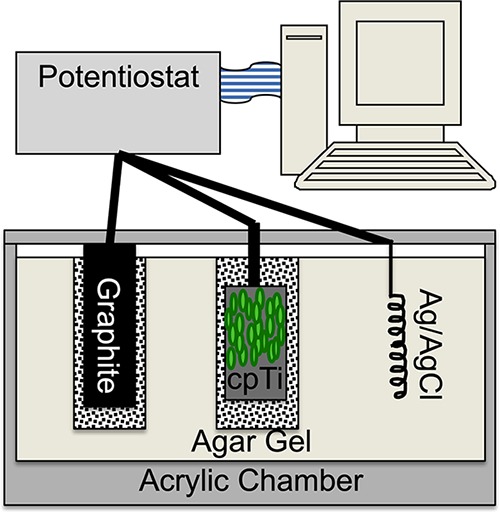
Schematic representing the custom*-*designed three-electrode potentiostatic electrochemical stimulation chamber. An eradication CVCES setup is depicted with a preformed biofilm (green) on the cpTi working electrode. (Reprinted from reference 18 with permission.)

Custom test chambers were fabricated by pouring hot liquid agar (1.5% pure agar) in sterile saline (0.9% NaCl, pH 7.2) into a sterile acrylic chamber (15, 18). Briefly, the agar chamber (Fig. 6) uses a standard three-electrode system with cpTi as the working electrode, a graphite counterelectrode, and an Ag/AgCl reference electrode. A graphite counterelectrode was placed into one of the two preformed wells, and the titanium working electrode was suspended in the remaining preformed well. The Ag/AgCl reference electrode was passed through the third port and embedded directly within the agar.

### Eradication studies: biofilm preparation.

Fresh cultures were suspended in medium to an optical density at 600 nm (OD_600_) of 0.05, corresponding to approximately 10^5^ CFU/ml, and then diluted 1:1,000 to determine the inoculum. The prepared titanium coupons were placed into 50-ml conical tubes containing 5 ml of the bacterial inoculum and incubated at 37°C for 18 h at 100 rpm to allow for the development of adherent biofilms on the submerged surface of the cpTi coupon (18). The titanium coupons coated with biofilm-associated bacteria were rinsed with sterile phosphate-buffered saline (PBS) to remove loosely adherent cells and then placed into the agar chamber as the working electrode. Subsequently, 3 ml of sterile medium with or without antibiotics, as required, was added to the working electrode well, and 3 ml of sterile medium was placed into the graphite counterelectrode well.

### Prevention studies: culture preparation.

Fresh cultures were inoculated into 5 ml of the appropriate broth medium at a 10:1 flask-to-volume ratio, adjusted to an OD_600_ of 0.1. Cultures were grown as described above at 180 rpm to mid-log phase, diluted back to an OD_600_ of 0.1 (corresponding to approximately 10^7^ CFU/ml), and then further diluted 1:10,000 in fresh medium for a starting inoculum of approximately 10^3^ CFU/ml. Each bacterial culture was evaluated in the presence and absence of the MIC of the appropriate antibiotic. Three milliliters of the test culture, with or without the strain-specific antibiotics, was added to the working electrode well, and 3 ml of sterile medium was placed into the graphite counterelectrode well.

### MIC.

Biofilms were prepared on titanium coupons as described above for the eradication studies and incubated with various concentrations of the antibiotic. After 24 h of incubation, biofilms were then dilution plated for CFU to determine the lowest concentration of the antibiotic required for eradication studies. Concentrations of 500 μg/ml vancomycin against NRS70 biofilms and 3,000 μg/ml gentamicin against PA27853 biofilms were determined. According to Clinical and Laboratory Standards Institute (CLSI) methods for antimicrobial susceptibility testing, the MICs of planktonic vancomycin and gentamicin were evaluated. Planktonic cultures were prepared to 10^3^ CFU/ml, as described above for prevention experiments, and treated with various concentrations of the antibiotic by broth microdilution. Vancomycin has an MIC of 0.5 μg/ml against planktonic NRS70, and gentamicin has an MIC of 3 μg/ml against planktonic PA27853 (51).

### Cathodic-voltage-controlled electrical stimulation.

With respect to an Ag/AgCl reference electrode, a voltage of −1.0 V, −1.5 V, or −1.8 V for 24 h was applied to the titanium working electrode at room temperature using a potentiostat (Interface 1000; Gamry Instruments) in the presence and absence of vancomycin for both prevention and eradication experiments. No prevention testing was performed at −1.8 V due to the significant reductions observed previously after CVCES at −1.8 V for 8 h (15). Two additional conditions, antibiotic alone (i.e., no CVCES) and no-treatment controls (i.e., no CVCES and no antibiotic), were also evaluated for each experiment. Therefore, each set of eradication or prevention experiments performed for a given CVCES magnitude consisted of four experimental conditions: no-treatment controls, drug alone, CVCES alone, or CVCES and antibiotics combined. Each set of experiments was repeated four independent times for each CVCES magnitude. All voltages in this study are reported with respect to Ag/AgCl.

For PA27853 prevention and eradication experiments, in addition to adding the antibiotic to the medium in the working electrode well, the agar surrounding each of the wells was supplemented with gentamicin (3 μg/ml or 3,000 μg/ml for prevention or eradication, respectively) under antibiotic-alone and CVCES-and-drug conditions. This was done to prevent the antibiotics from diffusing from the well into the agar gel due to a concentration gradient, as had been observed in separate preliminary experiments (data not shown). Vancomycin is a large molecule, and after testing, diffusion into the agar was not observed (data not shown). Therefore, in the NRS70 prevention and eradication experiments, vancomycin was added only to the medium in the working electrode well and was not added to the agar gel because diffusion was not observed.

### Enumeration of CFU.

After 24 h of incubation under the given experimental conditions, titanium coupons were washed in sterile PBS–0.1% saponin (Sigma-Aldrich, St. Louis, MO) and sonicated in 1 ml of sterile PBS–0.1% saponin for 10 min to release adherent bacteria from the surface. Surviving biofilm-associated CFU were enumerated by plating 100 µl of serial 10-fold dilutions onto Mueller-Hinton agar plates. The plates were incubated for 18 h at 37°C in 5% CO_2_. Surviving planktonic bacteria were enumerated from the cathode by collecting the medium surrounding the cpTi working electrode and plating serial dilutions as described above to determine CFU.

### pH measurements.

A pH electrode (Seven Easy InLab413; Mettler Toledo) was used to measure the pH of the medium surrounding the titanium working electrode following the 24-h incubation/stimulation protocol for all experiments.

### Charge transfer calculations.

Cathodic current measurements were collected for the duration of CVCES under all conditions and averaged across treatments with and without antibiotics. The cathodic current was integrated with respect to time to determine the total charge transferred during stimulation. Using a Riemann sum trapezoidal method, calculations were performed in Microsoft Excel to determine the cumulative charge transferred during CVCES. The cathodic current was area normalized by dividing by the surface area of the working electrode and reported as cathodic current density.

### Eradication and prevention pH models.

TSBG and MH broth were adjusted to pH 7, 8, 9, 10, 11, and 12 with the addition of NaOH or HCl, where appropriate, and filter sterilized prior to use. pH was confirmed using a Mettler Toledo Seven Easy InLab413 pH electrode.

In the biofilm pH model, fresh cultures were suspended to an OD_600_ of 0.05, corresponding to approximately 10^5^ CFU/ml. Cultures were diluted 1:1,000 to determine the inoculum. One milliliter of the prepared culture was used to seed the bottom of polystyrene 24-well plates, and biofilms were cultured at 37°C in 5% CO_2_ for 18 h. The supernatant was then removed and replaced with 3 ml of fresh medium adjusted to pH 7 to 12. Samples were incubated for an additional 24 h at room temperature without agitation. The supernatant was removed, and biofilms were collected from each well after mechanical resuspension in 1 ml of PBS. One hundred microliters of serial 10-fold dilutions was plated onto MH agar plates to enumerate surviving bacteria. The plates were incubated for 18 h at 37°C in 5% CO_2_. Each experiment consisted of three independent samples from two pooled wells.

For planktonic pH experiments, fresh cultures were inoculated at a 10:1 flask-to-volume ratio, adjusted to an OD_600_ of 0.1, and placed in a 37°C shaking water bath at 180 rpm. Cultures were grown to mid-log phase and diluted back to an OD_600_ of 0.1. Cultures were then further diluted 1:10,000 in the appropriate fresh medium at either pH 7, 8, 9, 10, 11, or 12, for a starting inoculum of approximately 10^3^ CFU/ml for each pH assay. Under each condition, 3-ml aliquots of the pH-adjusted bacterial inocula were added to individual wells of a 24-well polystyrene dish. Samples were incubated for 24 h at room temperature without agitation. After 24 h, medium in the well was collected. One hundred microliters of serial 10-fold dilutions was plated onto MH agar plates to enumerate surviving bacteria. The plates were incubated for 18 h at 37°C in 5% CO_2_.

### Statistical analysis.

One-way analysis of variance (ANOVA) followed by Tukey *post hoc* analysis, where appropriate, was used to compare the log-transformed CFU and pHs across treatment conditions (control, drug, CVCES, and CVCES with drug) for both eradication and prevention experiments. ANOVA was also used to compare the log-transformed current densities and charge transfers across stimulation conditions (−1.0 V, −1.5 V, and −1.8 V). Alternatively, if the Levene’s test was significant, the data were analyzed by Welch’s test followed by a Games-Howell *post hoc* test, where appropriate. For the pH incubation experiments, ANOVA followed by a Dunnett test was used to individually compare viable CFU from pH 7 to viable CFU from pH 8 to 12. In addition, Student’s *t* tests were used to compare the cathodic current densities and cumulative charge transfers for the prevention experiments under −1.0-V and −1.5-V conditions. A *P* value of 0.05 was considered significant in all tests. The statistical analysis was performed with SPSS statistical software, version 12 (IBM Corp., Armonk, NY).

## References

[1] Kurtz S, Ong K, Lau E, Mowat F, Halpern M 2007 Projections of primary and revision hip and knee arthroplasty in the United States from 2005 to 2030. J Bone Joint Surg Am 89:780–785. doi:10.2106/JBJS.F.00222. 17403800

[2] Kurtz S, Ong K, Schmier J, Mowat F, Saleh K, Dybvik E, Karrholm J, Havelin L, Ove F, Malchau H, Lau E 2007 Future clinical and economic impact of revision total hip and knee arthroplasty. J Bone Joint Surg Am 89:144–151. doi:10.2106/JBJS.G.00587. 17908880

[3] Costerton JW, Cheng KJ, Geesey GG, Ladd TI, Nickel JC, Dasgupta M, Marrie TJ 1987 Bacterial biofilms in nature and disease. Annu Rev Microbiol 41:435–464. doi:10.1146/annurev.mi.41.100187.002251. 3318676

[4] Boucher HW, Talbot GH, Bradley JS, Edwards JE, Gilbert D, Rice LB, Scheld M, Spellberg B, Bartlett J 2009 Bad bugs, no drugs: no ESKAPE! An update from the Infectious Diseases Society of America. Clin Infect Dis 48:1–12. doi:10.1086/595011. 19035777

[5] Santajit S, Indrawattana N 2016 Mechanisms of antimicrobial resistance in ESKAPE pathogens. Biomed Res Int 2016:2475067. doi:10.1155/2016/2475067. 27274985 PMC4871955

[6] Geipel U 2009 Pathogenic organisms in hip joint infections. Int J Med Sci 6:234–240. 19834588 10.7150/ijms.6.234 PMC2755118

[7] Del Pozo JL, Patel R 2009 Infection associated with prosthetic joints. N Engl J Med 361:787–794. doi:10.1056/NEJMcp0905029. 19692690 PMC2850113

[8] Rafati M, Shiva A, Ahmadi A, Habibi O 2014 Adherence to American Society of Health-System Pharmacists surgical antibiotic prophylaxis guidelines in a teaching hospital. J Res Pharm Pract 3:62–66. doi:10.4103/2279-042X.137075. 25114939 PMC4124682

[9] Dubrovskaya Y, Tejada R, Bosco J, Stachel A, Chen D, Feng M, Rosenberg A, Phillips M 2015 Single high dose gentamicin for perioperative prophylaxis in orthopedic surgery: evaluation of nephrotoxicity. SAGE Open Med 3:2050312115612803. doi:10.1177/2050312115612803. 26770808 PMC4679329

[10] Parvizi J, Pawasarat IM, Azzam KA, Joshi A, Hansen EN, Bozic KJ 2010 Periprosthetic joint infection: the economic impact of methicillin-resistant infections. J Arthroplasty 25:103–107. doi:10.1016/j.arth.2010.04.011. 20570103

[11] Zimmerli W 2006 Prosthetic-joint-associated infections. Best Pract Res Clin Rheumatol 20:1045–1063. doi:10.1016/j.berh.2006.08.003. 17127196

[12] Costerton JW. 2005 Biofilm theory can guide the treatment of device-related orthopaedic infections. Clin Orthop Relat Res 437:7–11. 10.1097/00003086-200508000-00003 16056019

[13] Zmistowski B, Karam JA, Durinka JB, Casper DS, Parvizi J 2013 Periprosthetic joint infection increases the risk of one-year mortality. J Bone Joint Surg Am 95:2177–2184. doi:10.2106/JBJS.L.00789. 24352771

[14] Brooks E, Tobias M, Krautsak K, Ehrensberger M 2014 The influence of cathodic polarization and simulated inflammation on titanium electrochemistry. J Biomed Mater Res B Appl Biomater 102:1445–1453. doi:10.1002/jbm.b.33123. 24610893

[15] Canty M, Luke-Marshall N, Campagnari A, Ehrensberger M 2017 Cathodic voltage-controlled electrical stimulation of titanium for prevention of methicillin-resistant Staphylococcus aureus and Acinetobacter baumannii biofilm infections. Acta Biomater 48:451–460. doi:10.1016/j.actbio.2016.11.056. 27890730

[16] Ehrensberger MT, Gilbert JL 2010 The effect of scanning electrochemical potential on the short-term impedance of commercially pure titanium in simulated biological conditions. J Biomed Mater Res A 94:781–789. doi:10.1002/jbm.a.32736. 20336755

[17] Ehrensberger MT, Gilbert JL 2010 The effect of static applied potential on the 24-hour impedance behavior of commercially pure titanium in simulated biological conditions. J Biomed Mater Res B Appl Biomater 93:106–112. doi:10.1002/jbm.b.31564. 20091908

[18] Ehrensberger MT, Tobias ME, Nodzo SR, Hansen LA, Luke-Marshall NR, Cole RF, Wild LM, Campagnari AA 2015 Cathodic voltage-controlled electrical stimulation of titanium implants as treatment for methicillin-resistant Staphylococcus aureus periprosthetic infections. Biomaterials 41:97–105. doi:10.1016/j.biomaterials.2014.11.013. 25522969

[19] Schneider S, Rudolph M, Bause V, Terfort A 2018 Electrochemical removal of biofilms from titanium dental implant surfaces. Bioelectrochemistry 121:84–94. doi:10.1016/j.bioelechem.2018.01.008. 29413867

[20] Nodzo SR, Tobias M, Ahn R, Hansen L, Luke-Marshall NR, Howard C, Wild L, Campagnari AA, Ehrensberger MT 2016 Cathodic voltage-controlled electrical stimulation plus prolonged vancomycin reduce bacterial burden of a titanium implant-associated infection in a rodent model. Clin Orthop Relat Res 474:1668–1675. doi:10.1007/s11999-016-4705-7. 26801677 PMC4887353

[21] Nodzo S, Tobias M, Hansen L, Luke-Marshall NR, Cole R, Wild L, Campagnari AA, Ehrensberger MT 2015 Cathodic electrical stimulation combined with vancomycin enhances treatment of methicillin-resistant Staphylococcus aureus implant-associated infections. Clin Orthop Relat Res 473:2856–2864. doi:10.1007/s11999-015-4280-3. 25825157 PMC4523523

[22] del Pozo JL, Rouse MS, Mandrekar JN, Steckelberg JM, Patel R 2009 The electricidal effect: reduction of Staphylococcus and Pseudomonas biofilms by prolonged exposure to low-intensity electrical current. Antimicrob Agents Chemother 53:41–45. doi:10.1128/AAC.00680-08. 18955534 PMC2612149

[23] Mohn D, Zehnder M, Stark WJ, Imfeld T 2011 Electrochemical disinfection of dental implants—a proof of concept. PLoS One 6:e16157. doi:10.1371/journal.pone.0016157. 21264247 PMC3021527

[24] Rabinovitch C, Stewart PS 2006 Removal and inactivation of Staphylococcus epidermidis biofilms by electrolysis. Appl Environ Microbiol 72:6364–6366. doi:10.1128/AEM.00442-06. 16957263 PMC1563645

[25] Sandvik EL, McLeod BR, Parker AE, Stewart PS 2013 Direct electric current treatment under physiologic saline conditions kills Staphylococcus epidermidis biofilms via electrolytic generation of hypochlorous acid. PLoS One 8:e55118. doi:10.1371/journal.pone.0055118. 23390518 PMC3563656

[26] Stinson MW 2009 Growth and nutrition of bacteria, p 46–47. *In* Thacore HR, Shanahan TC (ed), Microbiology & immunology for health related professionals, 3rd ed Pearson Custom Publishing, New York, NY.

[27] Pulido L, Ghanem E, Joshi A, Purtill J, Parvizi J 2008 Periprosthetic joint infection: the incidence, timing, and predisposing factors. Clin Orthop Relat Res 466:1710–1715. doi:10.1007/s11999-008-0209-4. 18421542 PMC2505241

[28] Siljander MP, Sobh AH, Baker KC, Baker EA, Kaplan LM 2018 Multidrug-resistant organisms in the setting of periprosthetic joint infection—diagnosis, prevention, and treatment. J Arthroplasty 33:185–194. doi:10.1016/j.arth.2017.07.045. 28869114

[29] Ravi S, Zhu M, Luey C, Young SW 2016 Antibiotic resistance in early periprosthetic joint infection. ANZ J Surg 86:1014–1018. doi:10.1111/ans.13720. 27561596

[30] Fulkerson E, Valle CJD, Wise B, Walsh M, Preston C, Di Cesare PE 2006 Antibiotic susceptibility of bacteria infecting total joint arthroplasty sites. J Bone Joint Surg Am 88:1231–1237. doi:10.2106/JBJS.E.00004. 16757755

[31] Liu WK, Brown MR, Elliott TS 1997 Mechanisms of the bactericidal activity of low amperage electric current (DC). J Antimicrob Chemother 39:687–695. doi:10.1093/jac/39.6.687. 9222036

[32] Sultana ST, Atci E, Babauta JT, Falghoush AM, Snekvik KR, Call DR, Beyenal H 2015 Electrochemical scaffold generates localized, low concentration of hydrogen peroxide that inhibits bacterial pathogens and biofilms. Sci Rep 5:14908. doi:10.1038/srep14908. 26464174 PMC4604468

[33] Sultana ST, Call DR, Beyenal H 2016 Eradication of Pseudomonas aeruginosa biofilms and persister cells using an electrochemical scaffold and enhanced antibiotic susceptibility. NPJ Biofilms Microbiomes 2:2. doi:10.1038/s41522-016-0003-0. 28649396 PMC5460242

[34] Babauta JT, Nguyen HD, Istanbullu O, Beyenal H 2013 Microscale gradients of oxygen, hydrogen peroxide, and pH in freshwater cathodic biofilms. ChemSusChem 6:1252–1261. doi:10.1002/cssc.201300019. 23766295 PMC4247834

[35] Atci E, Babauta JT, Beyenal H 2016 A hydrogen peroxide microelectrode to use in bioelectrochemical systems. Sens Actuators B Chem 226:429–435. doi:10.1016/j.snb.2015.12.004.

[36] Dargahi M, Hosseinidoust Z, Tufenkji N, Omanovic S 2014 Investigating electrochemical removal of bacterial biofilms from stainless steel substrates. Colloids Surf B Biointerfaces 117:152–157. doi:10.1016/j.colsurfb.2014.02.021. 24681392

[37] Blenkinsopp SA, Khoury AE, Costerton JW 1992 Electrical enhancement of biocide efficacy against *Pseudomonas aeruginosa* biofilms. Appl Environ Microbiol 58:3770–3773. 1482196 10.1128/aem.58.11.3770-3773.1992 PMC183173

[38] Costerton JW, Ellis B, Lam K, Johnson F, Khoury AE 1994 Mechanism of electrical enhancement of efficacy of antibiotics in killing biofilm bacteria. Antimicrob Agents Chemother 38:2803–2809. doi:10.1128/AAC.38.12.2803. 7695266 PMC188289

[39] del Pozo JL, Rouse MS, Mandrekar JN, Sampedro MF, Steckelberg JM, Patel R 2009 Effect of electrical current on the activities of antimicrobial agents against Pseudomonas aeruginosa, Staphylococcus aureus, and Staphylococcus epidermidis biofilms. Antimicrob Agents Chemother 53:35–40. doi:10.1128/AAC.00237-08. 18725436 PMC2612137

[40] Jass J, Costerton JW, Lappin-Scott HM 1995 The effect of electrical currents and tobramycin on Pseudomonas aeruginosa biofilms. J Ind Microbiol 15:234–242. doi:10.1007/BF01569830. 8519482

[41] Niepa THR, Gilbert JL, Ren DC 2012 Controlling Pseudomonas aeruginosa persister cells by weak electrochemical currents and synergistic effects with tobramycin. Biomaterials 33:7356–7365. doi:10.1016/j.biomaterials.2012.06.092. 22840233

[42] Niepa THR, Snepenger LM, Wang H, Sivan S, Gilbert JL, Jones MB, Ren D 2016 Sensitizing Pseudomonas aeruginosa to antibiotics by electrochemical disruption of membrane functions. Biomaterials 74:267–279. doi:10.1016/j.biomaterials.2015.10.007. 26461119

[43] Stoodley P, deBeer D, Lappin-Scott HM 1997 Influence of electric fields and pH on biofilm structure as related to the bioelectric effect. Antimicrob Agents Chemother 41:1876–1879. doi:10.1128/AAC.41.9.1876. 9303377 PMC164028

[44] Wellman N, Fortun SM, McLeod BR 1996 Bacterial biofilms and the bioelectric effect. Antimicrob Agents Chemother 40:2012–2014. doi:10.1128/AAC.40.9.2012. 8878572 PMC163464

[45] Baatout S, Leys N, Hendrickx L, Dams A, Mergeay M 2007 Physiological changes induced in bacteria following pH stress as a model for space research. Acta Astronaut 60:451–459. doi:10.1016/j.actaastro.2006.09.012.

[46] Gilman S, Saunders VA 1986 Accumulation of gentamicin by Staphylococcus aureus: the role of the transmembrane electrical potential. J Antimicrob Chemother 17:37–44. doi:10.1093/jac/17.1.37. 3949638

[47] Bartek IL, Reichlen MJ, Honaker RW, Leistikow RL, Clambey ET, Scobey MS, Hinds AB, Born SE, Covey CR, Schurr MJ, Lenaerts AJ, Voskuil MI 2016 Antibiotic bactericidal activity is countered by maintaining pH homeostasis in Mycobacterium smegmatis. mSphere 1:e00176-16. doi:10.1128/mSphere.00176-16. 27579369 PMC4999920

[48] Taber HW, Mueller JP, Miller PF, Arrow AS 1987 Bacterial uptake of aminoglycoside antibiotics. Microbiol Rev 51:439–457. 3325794 10.1128/mr.51.4.439-457.1987 PMC373126

[49] Allison KR, Brynildsen MP, Collins JJ 2011 Metabolite-enabled eradication of bacterial persisters by aminoglycosides. Nature 473:216–220. doi:10.1038/nature10069. 21562562 PMC3145328

[50] Kuroda M, Ohta T, Uchiyama I, Baba T, Yuzawa H, Kobayashi I, Cui L, Oguchi A, Aoki K, Nagai Y, Lian J, Ito T, Kanamori M, Matsumaru H, Maruyama A, Murakami H, Hosoyama A, Mizutani-Ui Y, Takahashi NK, Sawano T, Inoue R-I, Kaito C, Sekimizu K, Hirakawa H, Kuhara S, Goto S, Yabuzaki J, Kanehisa M, Yamashita A, Oshima K, Furuya K, Yoshino C, Shiba T, Hattori M, Ogasawara N, Hayashi H, Hiramatsu K 2001 Whole genome sequencing of meticillin-resistant Staphylococcus aureus. Lancet 357:1225–1240. doi:10.1016/S0140-6736(00)04403-2. 11418146

[51] CLSI. 2010 Performance standards for antimicrobial susceptibility testing; twentieth informal supplement M100-S20. CSLI, Wayne, PA.

